# When a Differential Diagnosis Is Fundamental: Choriocarcinoma Mimicking Lung Carcinoma

**DOI:** 10.3390/jcm8112018

**Published:** 2019-11-19

**Authors:** Roberto Gasparri, Giulia Sedda, Daniela Brambilla, Lara Girelli, Cristina Diotti, Lorenzo Spaggiari

**Affiliations:** 1 Department of Thoracic Surgery, IEO, European Institute of Oncology IRCCS, 20141 Milan, Italy; giulia.sedda@ieo.it (G.S.); lara.girelli@ieo.it (L.G.); cristina.diotti@gmail.com (C.D.); lorenzo.spaggiari@ieo.it (L.S.); 2 Department of Data Management, IEO, European Institute of Oncology IRCCS, 20141 Milan, Italy; daniela.brambilla@ieo.it; 3 Department of Oncology and Hemato-Oncology, University of Milan, 20139 Milan, Italy

**Keywords:** lung, neoplasm, choriocarcinoma, diagnosis

## Abstract

**Background:** Choriocarcinoma is a rare malignant disease that is usually associated with a gestational event. Lung metastasis with no evident primary origin and choriocarcinoma, which mimics features of non-small-cell lung cancer, might be misdiagnosed as adenocarcinoma or large-cell carcinoma. This is a pivotal clinical concern since the tumor can lead to various symptoms, seriously affecting the quality of life and can escalate rapidly, with a high mortality rate, compared to lung cancer. **Methods:** We reported a case of a 37-year-old woman with a history of one-year enhancement of beta-human chorionic gonadotropin levels and only a single nodule in the right upper lobe, with no abnormal findings on the gynecological investigation. Then we retrospectively examined all cases treated in the Division of Thoracic Surgery at the European Institute of Oncology in the last twenty years (from 1998 to 2018). **Results:** This was the first time in our experience that choriocarcinoma presentation was with a single nodule without a gynecological finding. Moreover, the differential diagnosis between lung carcinoma and choriocarcinoma was achieved only after surgical removal. **Conclusions:** As confirmed by our literature search, precise and expedited differential diagnosis is essential in choriocarcinoma care (both with single or multiple metastases), to successfully remove the tumor and increase the patient’s chances of survival.

## 1. Introduction

Choriocarcinoma is a very rare malignant disease that is usually associated with a gestational event, such as hydatidiform mole, spontaneous abortion, or ectopic pregnancy [1]. Non-gestational choriocarcinoma is remarkably rare, arising from pluripotent germ cells in the gonads or midline structures, such as the mediastinum [2]. Both of the classifications can have highly varied and atypical clinical presentation [3], most of the time with an uncertain origin [4,5,6].

The tumor is composed of cytotrophoblast (i.e., stem cell of the trophoblast lineage) and syncytiotrophoblast cells, which secrete beta-human chorionic gonadotropin (β-hCG). The tendency of choriocarcinoma toward early vascular invasiveness explains the high rate of metastasis mostly inside the lung (80–85% rate) but also in the vagina, brain, retroperitoneum, kidney, stomach, and small bowel [7,8]. Lung metastasis with unknown primary origin and choriocarcinoma, which mimics features of non-small-cell lung cancer, might be misdiagnosed as adenocarcinoma or large-cell carcinoma [9]. Only in 10% of the cases is the tumor found during a routine check-up in asymptomatic patients. In the remaining patients, it is usually discovered after the onset of symptoms, such as cough, chest pain or hemoptysis, which are manifestations in common with lung cancer. Moreover, often the differential diagnosis is obtained after tumor removal since initial biopsy does not allow diagnosis. Surgery is, therefore, the best choice to achieve diagnostic and curative intent in small resectable tumors. Oncological therapy consists of multimodality chemotherapy, including Bleomycin, Etoposide, and Cisplatin, along with radiation treatment.

## 2. Case Presentation

**Figure 1:**
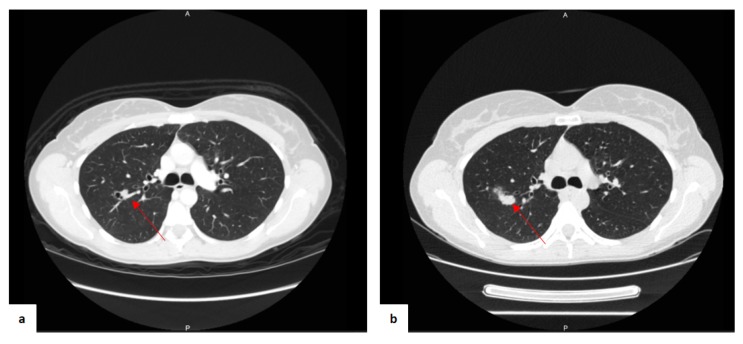
CT scan images of the first findings (**a**) and the findings after eight-month follow-up associated with antibiotic therapy (**b**). The nodule is indicated by the red arrow.

A 37-year-old woman was referred to our department, without any oncological history. The patient had two pregnancies followed by two natural childbirths (two daughters on 2013 and 2015, respectively), without any history of abortion or ectopic pregnancy. A routine blood exam executed because of the suspension of her monthly periods revealed high levels of β-hCG, but no pregnancies were detectable with echography investigation. She underwent a computed tomography (CT) scan and ^18^F-fluorodeoxyglucose (^18^F-FDG) positron-emission tomography (PET) investigation. The CT scan showed a single round nodule of 11 mm with net margins in the right lobe (Figure 1a).

**Figure 2:**
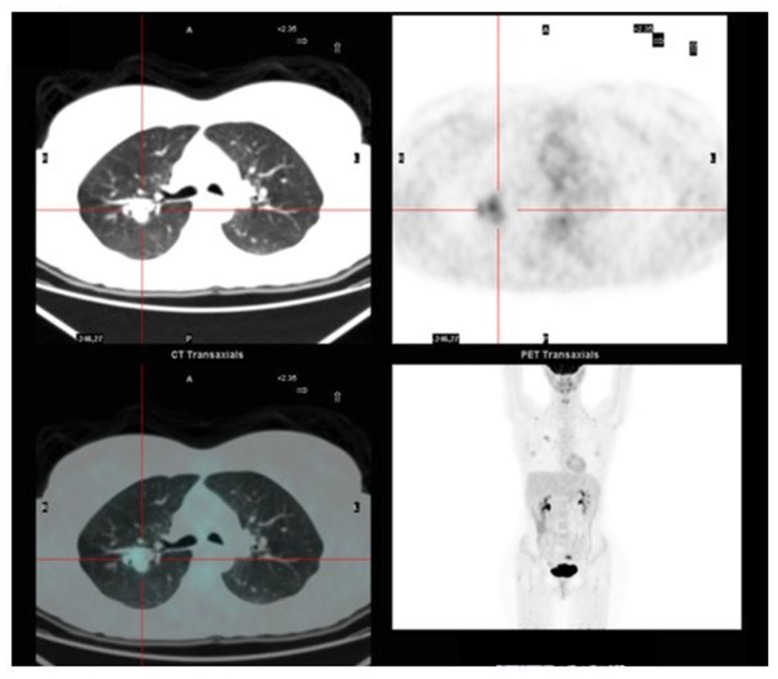
^18^F-FDG-PET of the pulmonary lesion.

The ^18^F-FDG-PET revealed glucose metabolic activity, not only in the nodule (Figure 2), but also in the left ovary.

Considering the nodule primary findings and onset of symptoms (i.e., episodes of severe cough and expectoration), she followed antibiotic therapy for 10 days with Amoxicillin with Clavulanic acid and Acetylcysteine, to differentiate the diagnosis between an inflammatory process and an oncological nodule. She underwent a repeat X-ray analysis, which confirmed the lesion without any variations. In the meantime, an ovarian biopsy was carried out, and no signs of malignancy, extrauterine pregnancy, or miscarriage were found.

**Figure 3:**
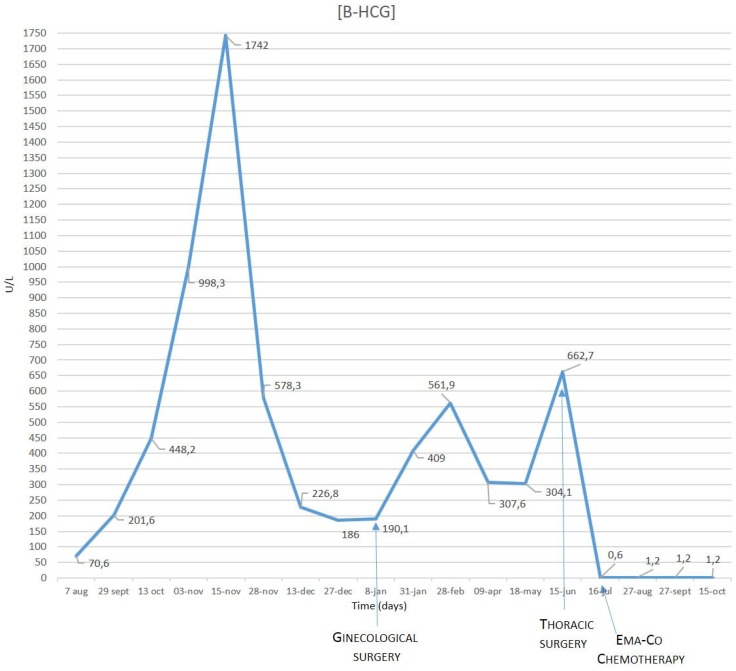
β-hCG level showed fluctuating concentrations during blood examinations.

At the six-month follow-up of the nodule with a CT scan, the pulmonary lesion with complex cystic-like morphology and a central excavation zone were increased in volume (Figure 1b). The β-hCG showed fluctuating concentrations during the monthly blood examinations (Figure 3). Other tumor markers, including carcinoembryonic antigen (CEA), squamous cell carcinoma antigen (SCC), and carbohydrate antigen (CA) 19-9, were within normal limits.

In order to obtain a diagnosis, the patient underwent an endobronchial ultrasound-guided transbronchial needle aspiration (EBUS-TBNA) procedure. After obtaining written informed consent from the patient, EBUS-TBNA was performed under fluoroscopic guidance, and the rapid on-site cytologic evaluation results were adequate. Additional transbronchial biopsy for bacterioscopic and Mycobacterium tuberculosis research was performed. No adverse events occurred during or immediately after the diagnostic procedure, and no complications were observed. The final histopathological analysis was inconclusive, and the pathologist identified only debris and necrotic materials associated with chronic and acute inflammation, as well as histiocytic deposits of hemosiderin, without evidence of malignancy. The search for resistant alcohol–acid bacilli by Ziehl–Neelsen coloration was negative. The only finding was alpha-hemolytic streptococci; the patient underwent treatment with ceftriaxone sodium and levofloxacin, to eradicate the infection.

At the multidisciplinary discussion, a two-month follow-up after the antibiotic treatment was suggested due to nodule dimensions and symptoms. The blood examination showed infection resolution, and the CT-scan confirmed the nodular lesion of 21 × 19 mm. Moreover, the patient underwent a 68Gallium DOTANOC PET, a positivity for neuroendocrine tumor. The results showed only a moderate receptor somatostatin receptor 2–5 type density enhancement.

Considering the persistence of the lesion and the increasing dimension and imaging results, together with the patient’s willingness to remove the nodule, a surgical procedure of the right upper lobe was scheduled, with diagnostic and curative intent. Right upper lobectomy with mediastinal radical lymphadenectomy was performed with an intraoperative diagnosis of the presence of neoplastic malignant cells (i.e., non-small-cell carcinoma). The patient was discharged after three postoperative days, without any complications and with good general conditions, apyretic and eupnoeic.

**Figure 4:**
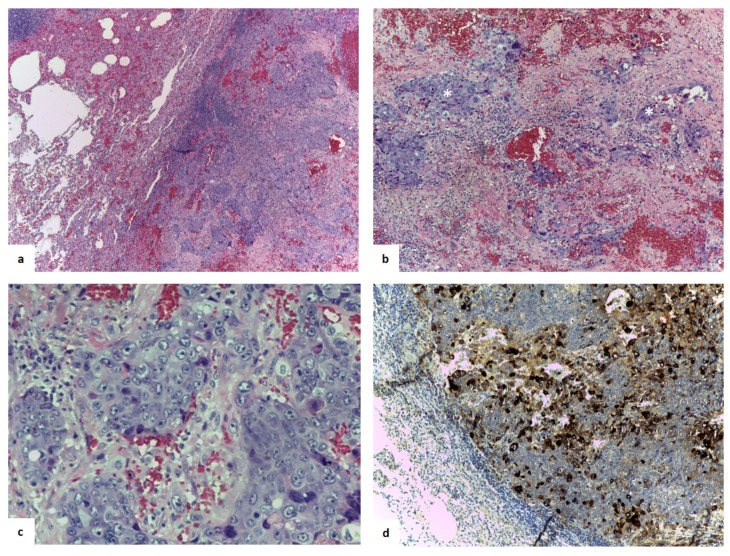
(**a**) Hematoxylin (blue staining of the nuclei) and eosin (pink staining of cytoplasm and extracellular matrix) mark showed a highly hemorrhagic tumor. (**b**) An intimate admixture of atypical cytotrophoblasts and syncytiotrophoblasts arranged in nests without a villus structure (enlighten with asterisks), magnified in (**c**). (**d**) The immunohistological findings indicated that the tumor cells were strongly positive (brown) for human chorionic gonadotropin (magnification in **a**: 10×, **b**,**c**, and **d**: 20×).

Immunohistochemically, the analysis was positive for cytokeratin 7 and AE1/AE3, p40, transformation-related protein 63, trophoblastic hormone, CD10, and β-hCG, and negative for inhibin and thyroid transcription factor-1 (Figure 4). The pathologist identified highly hemorrhagic features; visceral pleura and lymph nodes examined were free of neoplasia. The final diagnosis obtained was choriocarcinoma metastasis.

A multidisciplinary discussion considering the medical history of the young female patient with antecedent pregnancies and symptom origins was in favor of a lung metastatic gestational choriocarcinoma origin as being more likely than a lung primary choriocarcinoma or non-gestational choriocarcinoma.

In light of the diagnosis, the patient was referred to a gyneco-oncologist who, considering the histology and the high-risk score (FIGO staging III), opted for chemotherapy, according to the EMA-CO scheme, for two months (i.e., Etoposide, Methotrexate, Dactinomycin/Cyclophosphamide, and Vincristine).

At the two-year follow-up, the patient was in good condition, her monthly periods had come back, and normalization of the β-hCG serum level was observed. The clinical examination showed regular outcomes of surgery and optimal lung expansion, with no recurrence of the disease.

## 3. Retrospective Analysis

**Figure 5:**
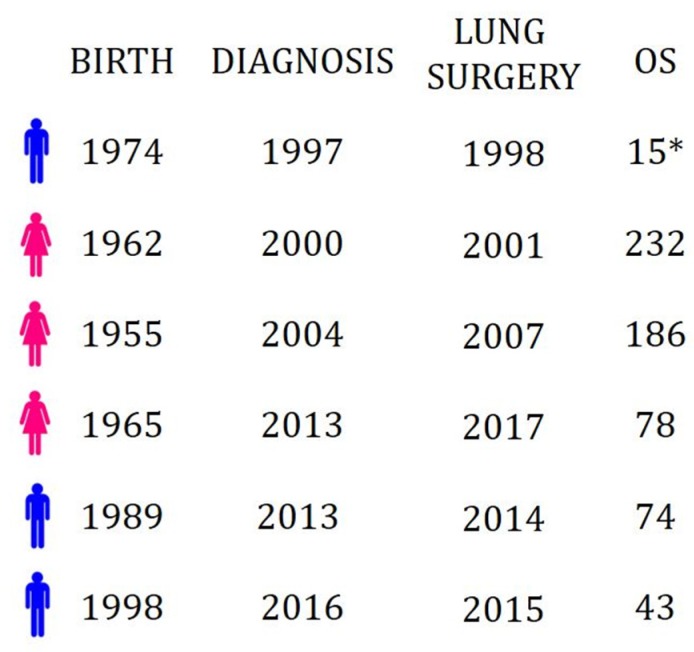
Descriptive timeline of the six cases of choriocarcinoma operated in the last 20 years at our institute.

Starting from the abovementioned case of single lung metastasis from choriocarcinoma, we retrospectively examined all cases treated in our division in the last twenty years (from 1998 to 2018). There were only six cases of choriocarcinoma lung metastasis (Figure 5), and all the patients’ clinical data are summarized in Table 1.

The Ethical Committee approved data collection and analysis and waived the need for written consent, since this was a retrospective analysis.

The differential diagnoses for the other six cases were two lung choriocarcinoma metastases related to a hydatidiform mole, two mediastinal anterior masses composed of choriocarcinoma mixed with other germ cell tumors (mature teratoma and a dysgerminoma), two choriocarcinoma metastasis from, respectively, primary disease uterus and testicles. The major difference was that the primary origin of these metastases was identifiable before lung surgery, thanks to conventional diagnosis. All the patients showed a higher level of β-hCG and received chemotherapy before and/or after surgery.

Among the six cases, only one patient died at 24 years (Patient 1 in Table 1): he had a diagnosis of testicular choriocarcinoma for which he underwent a right orchifunicolectomy, followed by multiple lines of chemotherapy. The disease was resistant to chemotherapy and progressed into several multiple metastases. The patient underwent atypical double resection of the lingula, diaphragm, pericardium, two pulmonary nodules, and extrapleural formation of the vertebral cost shower with lymphadenectomy to remove different metastases. He died 15 months after the original diagnosis.

The other patients are alive, with no evidence of recurrence. The longest follow-up is a female patient with multiple pulmonary nodules removed successfully 19 years ago and with complete disease remission.

## 4. Discussion

Among the different choriocarcinoma distant recurrence sites, the lung is the most frequent site for metastasis and is associated with lethal hemorrhagic complications [1]. Indeed, in the published literature, the prognosis is reported as poor due to the rapidity of tumor growth and association of bleeding risk with surgery, barotrauma, and acute respiratory distress syndrome.

In twenty years at our institute, which is a high-volume center of thoracic oncology, seven patients presented choriocarcinoma metastases in the thoracic region. Three men were characterized with testicular or mediastinal masses which generated metastasis inside the lung parenchyma (non-gestational choriocarcinoma). Four women had two hydatidiform moles or female-organ origins. Typically, histology was positive for trophoblastic hormone and associated with high β-hCG levels.

Among all the cases, the one that we report here is particularly challenging, since the patient had no previous diagnosis achieved before lung surgery and presented an alteration of the menstrual period, without any associated pregnancy. Imaging analysis showed a single round nodule with net margins in the right lobe, positive for glucose uptake and suggestive of lung cancer primary tumor. Moreover, usual approaches, such as EBUS-TBNA or percutaneous needle biopsy, for lung cancer staging were inconclusive. β-hCG-producing large or giant cell carcinoma of the lung is not uncommon, and the differential diagnosis with choriocarcinoma is essential to treat the patient efficiently. In the literature, only a few cases were reported with similar tumor features (i.e., no primary tumor detectable but only one lung single metastasis), which are reported in Table 2.

## 5. Conclusions

Choriocarcinoma lung single metastasis is a diagnostic challenge with standard methods without a surgical approach and a histopathological definitive differential investigation. This is a pivotal clinical concern since the tumor can lead to various symptoms, seriously affecting the quality of life, and it can escalate rapidly and has a high mortality rate [16]. Surgery represents the best choice to achieve diagnostic and curative intent when the choriocarcinoma is localized and resectable. Combining surgery with a chemotherapy regimen can improve therapeutic efficacy. Β-hCG level monitoring, together with symptom evaluation, can be useful as a tumor marker for following-up with the tumor and spotting any recurrence of the disease.

In conclusion, we report a case of lung metastasis from choriocarcinoma different from the other cases we have treated so far without diagnosis achieved with the preoperative clinical investigation. The follow-up of our patients showed that six of our patients are alive, in good health, and without any signs of recurrence, highlighting the fact that a precise and expedited diagnosis is essential in choriocarcinoma care (both with single or multiple metastases), in order to successfully remove the tumor and increase the patient’s chances of survival.

## Figures and Tables

**Table 1:** Patient clinical data and characteristics.

	Sex	Age	Origin	Chemotherapy	Type of Chemotherapy	OS (months)
Patient 1	M	24	Testicular	Post-surgery	Different lines	15 *
Patient 2	F	52	Uterus	Before surgery	Different lines	209
Patient 3	F	52	Hydatidiform mole	Before and post-surgery	Methotrexate and Actinomycin-D (before); EMA-CO scheme (post)	139
Patient 4	M	17	Mediastinal anterior masses	Before surgery	Bleomycin, Etoposide, Cisplatin (BEP scheme)	32
Patient 5	F	52	Hydatidiform mole	Before and post-surgery	Methotrexate and EMA-CO scheme	9
Patient 6	M	25	Mediastinal anterior masses	Before surgery	Bleomycin, Etoposide, Cisplatin (BEP scheme)	58

Age: at the first diagnosis; chemotherapy: before and after lung surgery; OS: overall survival from the first diagnosis; *: dead.

**Table 2:** Literature review of single lung metastasis report from choriocarcinoma. All patients were female.

Ref	Age	Hypothesized Origin	Treatment	OS (months)
[10]	26	Hydatidiform mole 6 years early	Surgery	not reported
[11]	27	Gestational	Surgery	19
[12]	29	Gestational	Surgery + Chemotherapy 3	12
[13]	36	Gestational	Chemotherapy 1 + Surgery + Chemotherapy 2	8
[14]	42	Gestational	Surgery + Chemotherapy 2	6
[15]	45	Hydatidiform mole 6 years early	Surgery + Chemotherapy 1	87

Age: at the first diagnosis; hypothesized origin: the supposed origin of choriocarcinoma; chemotherapy 1: Methotrexate; chemotherapy 2: EMA-CO scheme; chemotherapy 3: Etoposide, Methotrexate, Actinomycin, and Cisplatinum; OS: overall survival from the first diagnosis.

## Abbreviations

beta-human chorionic gonadotropin	β-hCG
computed tomography	CT
^18^F-fluorodeoxyglucose	^18^F-FDG
positron-emission tomography	PET
endobronchial ultrasound-guided transbronchial needle aspiration	EBUS-TBNA
etoposide methotrexate and dactinomycin/cyclophosphamide and vincristine	EMA-CO schemed
